# Zika virus: Epidemiology, current phobia and preparedness for upcoming mass gatherings, with examples from World Olympics and Pilgrimage

**DOI:** 10.12669/pjms.324.10038

**Published:** 2016

**Authors:** Nahla Khamis Ibrahim

**Affiliations:** 1 Prof. Nahla Khamis Ibrahim, MBBCh, MPH, Dr.PH, DHPE, JMHPE. Professor of Epidemiology, Family & Community Medicine Department, King Abdulaziz University, Jeddah, Saudi Arabia. Epidemiology Department, High Institute of Public Health, Alexandria University, Alexandria, Egypt

**Keywords:** Emerging diseases, Zika, Epidemiology, Phobia, Mass gathering, Vaccine trials, Preparedness, Surveillance

## Abstract

**Objective:**

To describe Zika Virus (ZIKV) epidemiology, current phobia, and the required preparedness for its prevention during the upcoming Mass Gathering (MG) events.

**Methods:**

Electronic databases of PubMed, WHO, CDC, Pan American Health Organization (PAHO), Google, and Cochrane library were extensively searched for ZIKV. Articles were reviewed, scrutinized and critically appraised and the most relevant articles were utilized.

**Results:**

ZIKV is an emerging Flavivirus which was first isolated from Uganda in 1947. It is transmitted mainly through bite of Aedes mosquitoes. Sexual, perinatal and blood-borne transmissions are implicated. ZIKV is incriminated to cause microcephaly and Guillain-Barré syndrome. The spiky spread of ZIKV and its epidemic potential are especially problematic in countries which host big MGs with endogenous ZIKV circulation. This put millions of international travelers and local inhabitants at risk of acquiring ZIKV, especially in absence of vaccine until now. Brazil Olympic and Paralympics Games, and Muslims Hajj in Saudi Arabia are important upcoming MGs. Regarding Brazil, swiftly epidemic of ZIKV causes phobia and provokes claims and counter-claims about possible postponing or cancellation of such events.

**Recommendations:**

Intensifying ZIKV epidemiological surveillance (sentinel, syndromic, environmental, laboratory and electronic), and conduction of educational programs are required. Controlling Aedes vector (chemically & biologically) is essential. Multidisciplinary cooperation is required to win the war against ZIKV.

## LITERATURE REVIEW

Nowadays, we are living in an era of eco-epidemiology;^1^ with global emergence and re-emergence of many Communicable Diseases (CDs). There is an unpredictable nature of the new pathogens. MERS-CoV, Khumra, Lassa, Influenza viral strains (H1N1, H5N1, H7N9, H10N8, etc.), Ebola, and ZIKV are some examples.^2^,^3^ Although Zika is very important and currently problem, until now inadequate comprehensive reviews were conducted regarding epidemiological preparedness for MGs. So, such review is needed.

The aim of this manuscript was to describe the Zika Virus epidemiology, current phobia, and the required preparedness for its prevention during the upcoming Mass Gathering (MG) events.

### Methods

Electronic databases of PubMed, WHO, CDC, Pan American Health Organization (PAHO), Google, and Cochrane library were extensively searched for ZIKV and mass gathering. It was done from 1947 till May 2016. The keywords emerging diseases, Zika virus, epidemiology, phobia, mass gathering, preparedness, surveillance were searched. Many articles were reviewed, scrutinized and critically appraised and the most relevant articles were utilized.

## RESULTS

The WHO declared ZIKV a “Public Health Emergency of International Concern (PHEIC)”, with triggering funding into research, vector control, and efforts to stop pregnant women becoming infected.^4^ In the following part of the manuscript the epidemiology, phobia against ZIKV in mass gathering and recommendation for its prevention and control is being discussed.

### Descriptive epidemiology of ZIKV

1. ***Person*** Approximately two-thirds of the world population lives in areas infested by *Aedes* (*Ae*.) mosquito vector.^5^ Furthermore, anyone who lives in or travels to an areas where ZIKV is already circulating and has not been infected with the virus can get it through mosquito bites. Once a person has been infected, he or she is likely to be protected from future infections. In addition, recently it was found that persons can catch infection through person to person transmission.^6^
2. ***Time*** The breading season of *Ae*. mosquitoes is usually during warm and rainy seasons in tropics and subtropics. The mosquito usually bite during the day times (after sunrise and before sunset).^6^
3. ***Place*** ZIKV was first isolated from Rhesus monkey in Zika Forest of Uganda, 1947. ZIKV was known to infect humans from Uganda and Nigeria based on serological surveys during 1952. Sporadic human cases were reported then from some African countries and Indonesia at the end of the 70’s. Epidemic was described in the Pacific in 2007 and 2013 (Yap and French Polynesia, respectively), and Cook Island and New Caledonia in 2014.^7^ During May 2015, the WHO notified the first local transmission of ZIKV in the Americas, with circulation in Brazil.^8^ Then the epidemic spread to other parts of South and North America and several islands in the Pacific. Since then, millions of infected individuals have been identified in Brazil, Colombia, Venezuela and other countries in the Americas.^9^ WHO reported on May 2016 that 60 countries and territories (mostly in Central and Latin Americas) have continuing mosquito-borne transmission. It was found that 46 countries are experiencing a first outbreak of Zika virus since 2015, with no previous evidence of circulation, and with ongoing transmission by mosquitoes. Another 14 countries reported evidence of Zika virus transmission between 2007 and 2014, with ongoing transmission.^6^ In the USA, it was reported that higher poverty rates of inhabitants in cities along the U.S.-Mexico border may be associated with increasing person exposure to Aedes aegypti.^10^ Millions of other new cases are expected to occur within the next 12 months.^9^

### Causation of ZIKV (analytic epidemiology

1. ***Agent*** ZIKV is an emerging 11-kb single stranded RNA arbovirus member of the *Flaviviridae family*, genus Flavivirus. It is related to Dengue fever, Yellow Fever, West Nile fever, Japanese encephalitis, and Chikungunya viruses.^7^,^11^
2. ***Host*** The vertebrate hosts of ZIKV were primarily monkeys in an “enzootic mosquito-monkey-mosquito cycle”, with only occasional transmission to humans. Before the current pandemic began in 2007, Zika rarely caused recognized ‘spillover’ infections in humans, even in highly enzootic areas. In the current pandemic, human is a host of ZIKV and local transmission between human has been reported in many countries and territories 6,12,13 Zika antibodies were detected also in ducks, goats, cows, horses, bats, and carabaos.^13^ The vector reservoir host is *Aedes* mosquitoes.^6^
3. ***Environment*** *Ae. aegypti* and *albopictus* live and lay eggs in the stagnant water collections and water-holding containers (as puddles, buckets, empty cans) around the households. Example of such areas are the peri-domestic areas with absent of the piped water provision; as in the slums in tropics and subtropics. This need warm and rainy season of the tropics and sub-tropics.^7^,^13^

### Modes of Zika virus transmission

1. ***Vector- borne (mosquito -borne) transmission*** ZIKV is chiefly transmitted by bite of the female.^7^ The main vector is *Ae. aegypti*, and it can also transmitted through other Aedes species as “*Ae. albopictus, Ae. africanus, Ae. luteocephalus, Ae. vitattus, Ae. furcifer, Ae. hensilii and Ae. apicoargenteus*”.^7^
2. ***Sexual transmission*** In 2014, ZIKV was proven to be capable of reproducing itself, and it was found in the semen of a man at least two weeks (may be up to 10 weeks) after he became ill with it.^14^ In May 2016, ten countries have reported evidence of person-to-person transmission of Zika virus, probably via a sexual route.^6^
3. ***Perinatal transmission*** In 2016, a proven evidence for causality was found between microcephaly, and related brain anomalies in infants, with the confirmed congenital ZIKV transmission.^15^ In Brazil, the incidence of microcephaly was also increased 20 times in 2015 compared to the previous years.^16^ ZIKV virus RNA was isolated from the brain tissue from the newborns, the placenta and other tissue of miscarriages of pregnant females who had foetuses with microcephaly in Brazil also.^17^ In mid 2016, microcephaly and other central nervous system (CNS) malformations associated with ZIKV congenital infection have been reported from 10 countries or territories. It was suggested also that ZIKV can be transmitted through breast feeding.^18^ ZIKV infection is suggested to cause also abortion and miscarriage.^17^
4. ***Blood-borne transfusion*** Given that the majority of cases with ZIKV infection are asymptomatic, and among them are some blood donors, transmission of ZIKV via blood transfusion is suggested during acute phase of infection.^7^
  On the other hand, other modes of transmission as saliva are still under studies.^14^

### Clinical picture and diagnosis

Most of the ZIKV cases are either in-apparent infections (about 80%) or mild ‘dengue-like’ syndrome.^14^ Symptomatic case usually presents with abrupt onset of mild fever, joint pain, headaches, retro-orbital pain, maculo-papular rash usually with itching, and conjunctivitis. Oedema of extremities, vertigo, myalgia and digestive disorder may also occur. These symptoms are usually mild and last for 2-7 days and the incubation period ranged 3-12 days.^7^,^19^ The cause of phobia about Zika is due to its suspected implication with increased neonatal microcephaly (as discussed), and other neurological conditions as Guillain-Barré syndrome.^4^,^7^ **Laboratory diagnosis**, Infection with ZIKV can be diagnosed by PCR or by IgG and IgM antibodies detection.^7^,^20^

### Mass gathering and phobia from Zika virus

Mass gatherings (MGs) may be big sports occasion, religious events, political or cultural events, and rock concerts.^21^,^22^ It occur worldwide on any given day, and Mass-Gathering Health (MGH) is a relatively new discipline.^23^ MGs have diverse public health challenges and need more international considerations, especially in presence of many emerging & re-emerging diseases and other health problems. This occurs in era of widely accessible air travel, and the world globalization.^24^ These events can put millions of international travellers and local host-country residents at risk of acquiring many infectious diseases, including locally endemic emerging infectious diseases as ZIKV.^25^

### Future mass gatherings in Americas (example from Brazil)

This spiky spread of ZIKV in Americas, especially in Brazil, poses both phobia and challenges during preparing for both the Olympics and Paralympics games which will be held in Rio De Janeiro during August and September, 2016 and also all future mass gathering that will take place in similar highly infectious countries.^7^,^20^ Brazil is working and needs to work more to protect more than 16,000 athletes and 600,000 visitors from catching ZIKV infections. There are claims and counter-claims about the suggested cancellation of these big occasions, after the big budget spent for preparing such events. Some countries have expressed fear about attending the MG events.^7^ However, the Brazilians’ officials said that among the factors that may decrease this phobia is that August is in the dry season, with less incidence of the mosquito that usually lays its eggs on stagnant water. Furthermore, Brazil is making efforts now in their war for controlling the mosquito and ZIKV before the big sport occasions, and similar upcoming occasions.

### Mass Gathering and Kingdom of Saudi Arabia (KSA)

Another example of the largest annual MGs is Muslim Hajj in KSA. More than 7 millions come every year to the KSA for Hajj and Omra.^25^,^26^ Until now there is no confirmed ZIKV cases in the KSA. However, preparedness of such MGs is required in the era of globalization and international travelling. Another reason for such preparedness is the presence of *Aedes* mosquito, and a similar *Arbovirus* which is Dengue fever virus in KSA.^5^ Standard measures applied every year prevent many diseases that can occur during Hajj and Omra.^7^,^20^ Collaborative efforts are also needed this year to prevent transmission of the ZIKV into KSA.

## FUTURE PERCEPTIVE, RECOMMENDATIONS AND PREPAREDNESS FOR MASS GATHERINGS

1. ***Health education*** All international airports and ports need to display educational materials for travelers about ZIKV, and how to avoid infection.^27^ In the heavily infected countries, Ministries of Health need to spread ZIKV information in streets and through media. It can include information in televised press, video-conferences and through social media (Twitter, Face book, YouTube, Instagram, Twitter podcasts, etc.). The CDC currently distributes ZIKV updated educational information through social media; and this can help travellers and local inhabitants from epidemic areas to avoid infection.^27^ Micro-websites with detailed information about ZIKV infection are established as the “CDC Zika Travel Information” website”.^27^ This will educate the public about how to remove, destroy and manage mosquito vector larval hebetates (stagnant want), and how prevent infection by avoiding mosquito bites (mosquito nets and repellent).^14^
2. ***Intensifying epidemiological surveillance*** It should be done with training of trainers on “preparedness and control of ZIKV and other emerging infectious diseases”. Training can be of different types of surveillance including routine, passive, active, environmental (Geographic Information System “GIS” for *Ae* mosquito breeding sites), sentinel, syndromic, laboratory and electronic surveillance. An Alert surveillance system for ZIKV will lead to immediate notification of the discovered cases, accurate determination of the magnitude of the problem and the high risk groups and hence application of preventive and control measures.^2^,^28^ These epidemiological measures are urgently needed for all countries infested with *Aedes* mosquito, especially in countries which will host big MGs as Brazil (with rapidly circulating ZIKV infection and heavy infestation with *Aedes* mosquito).^29^
3. ***Developing and monitoring global MG-specific integrated surveillance and alert systems*:**^29^ These measures are needed for ZIKV and other emerging infectious diseases. Success of such MG surveillance system will help to monitor the circulation of ZIKV in the host, and home countries (with varying capacity and resources of these countries). 28 Development and assessment of models for integrated risk management for local and international mass gatherings (MGs) is also required.^29^
4. ***Establishment and maintaining of “Zika Active Pregnancy Surveillance System” and prevention of perinatal transmission*** This surveillance need to be done in all countries with the circulating virus. ZIKV-infected pregnant females and their offspring need to be monitored for discovering any adverse maternal, fetal, neonatal, infant, and child health outcomes.^30^ Females from infected areas can post-pone pregnancy until more is known about relation of ZIKV to infants’ microcephaly, abortion and miscarriage. Testing is recommended at the start of antenatal care, with follow-up testing for asymptomatic pregnant women in infected areas with ZIKV.^30^,^31^ Furthermore, males who have a pregnant wife who live in or lately traveled to an active ZIKV transmission country need to abstain from sex or use condoms, during the pregnancy.^17^
5. ***Establishment and maintaining Travel Medicine*** Persons with immunodeficiency, severe chronic illnesses, and pregnant females, who plan to travel to the areas with transmission of ZIKV, need to discuss their travel plans with their physicians. They need to consider postponing their traveling to the areas with increasing or widespread transmission.^20^ In case that travelers are returning from affected areas, they need to report their suffering from any febrile illness, and need to be examined at airport (thermal Cameras).^14^
6. ***Personal Protection*** According to CDC recommendations, persons residing in or traveling to countries with active ZIKV transmission should prevent infection through prevention of mosquito bites. This can be done by wearing long-sleeved cloths, staying in places with air conditioning, using mosquito nets, mosquito repellents and treating clothing with insecticides.^28^ Anyone who has Zika infection should avoid mosquito bites during the first week of illness to protect others from getting sick through this mosquito.
7. ***Environmental sanitation:***
  - Permanent elimination of water containers by maintaining adequate supplies of piped water.^28^
  - ***Larvicides:*** chemicals or biological control to prevent the development of mosquito stages.^28^
  - ***Chemical control:*** for adult mosquitoes by space, residual and barrier spraying, and attractive toxic baits.^28^
  - ***Physical control (non-insecticidal mosquito traps):*** Gravid female mosquitoes can be attracted to traps baited with oviposition medium and captured using sticky glue while attempting to lay eggs. Use GIS and ovitrap can control the mosquito and decrease ovum.^28^,^32^
8. ***Treatment*** Until now there is no specific treatment, sero- prophylaxis, or chemo-prophylaxis for ZIKV. Symptomatic treatment can be done by adequate rest, sufficient fluids, medication to reduce fever and pain (as acetaminophen). Patients should avoid aspirin or other non-steroidal anti-inflammatory drugs.^31^
9. ***Disinfections*** WHO has recommended using aircraft disinfestations insecticides.^34^
10. ***Financial support and commitment*** It will be critical for controlling ZIKV and enhancing alertness of the surveillance system. Today, nearly two billion dollars has been requested from the U.S. Federal government to combat the exploding ZIKV pandemic.
11. ***Multidisciplinary international cooperation*** is required for controlling ZIKV epidemic. At national levels, especially in the countries infested with *Aedes* mosquito, cooperation is needed between governmental health, private sector, agriculture, natural resources, education, Non-Governmental Organizations (NGOs) and others to control mosquito vector.^33^
12. ***Future vaccination*** Until now, there is no vaccine against ZIKV. Various companies and educational institutes are now trying different vaccine approaches, as inactivated virus, virus-like particles, nucleic acid based vaccines, live vectored vaccines, subunit vaccines, and live recombinant approaches. Most of these are in preclinical trial development and many are expected to enter Phase I clinical trial in 2017.^35^
13. **Intensifying compulsory vaccination against yellow fever** This is needed for travelers to areas infested with *Aedes Aegypti mosquito*, especially for Brazil during the upcoming sport MGs.
14. **Control measures are needed for similar *Arbovirus*** as dengue, yellow fever, and Chikungunya.
15. **Research and development of new products:** Examples of such products are those for rapid diagnosis, vaccines and therapeutic interventions.
